# Five-week music therapy improves overall symptoms in schizophrenia by modulating theta and gamma oscillations

**DOI:** 10.3389/fpsyt.2024.1358726

**Published:** 2024-03-05

**Authors:** Lujie Wang, Liju Wang, Jiaxian Chen, Chenxi Qiu, Ting Liu, Yulin Wu, Yan Li, Pengyu Zou, Sijia Guo, Jing Lu

**Affiliations:** ^1^ Music and Digital Intelligence, Key Laboratory of Sichuan Province, Sichuan Conservatory of Music, Chengdu, China; ^2^ Department of Musicology, Sichuan Conservatory of Music, Chengdu, China; ^3^ Southwest Music Research Center, Key Research Base of Social Sciences in Sichuan Province, Sichuan Conservatory of Music, Chengdu, China; ^4^ Ministry of Education (MOE) Key Lab for Neuroinformation, School of Life Science and Technology, University of Electronic Science and Technology of China, Chengdu, China; ^5^ Department of Rehabilitation, Chengdu Dekang Hospital, Chengdu, China; ^6^ Yueling Music Therapy Service Center, Chengdu, China

**Keywords:** schizophrenia, music therapy, EEG, power spectral density, neural oscillations

## Abstract

**Introduction:**

Some clinical studies have shown that music therapy as an adjunctive therapy can improve overall symptoms in patients with schizophrenia. However, the neural mechanisms of this improvement remain unclear due to insufficient neuroimaging evidence.

**Methods:**

In this work, 17 patients with schizophrenia accepted a five-week music therapy (music group) that integrated listening, singing, and composing, and required patients to cooperate in a group to complete music therapy tasks. Meanwhile, 15 patients with schizophrenia received a five-week visual art intervention as the control group including handicraft and painting activities. We collected the Manchester Scale (MS) and Positive and Negative Symptom Scale (PANSS) scores and electroencephalography (EEG) data before and after intervention in two groups.

**Results:**

Behavioral results showed that both interventions mentioned above can effectively help patients with schizophrenia relieve their overall symptoms, while a trend-level effect was observed in favor of music therapy. The EEG results indicated that music therapy can improve abnormal neural oscillations in schizophrenia which is reflected by a decrease in theta oscillation in the parietal lobe and an increase in gamma oscillation in the prefrontal lobe. In addition, correlation analysis showed that in the music group, both reductions in theta oscillations in the parietal lobe and increases in gamma oscillations in the prefrontal lobe were positively correlated with the improvement of overall symptoms.

**Discussion:**

These findings help us to better understand the neural mechanisms by which music therapy improves overall symptoms in schizophrenia and provide more evidence for the application of music therapy in other psychiatric disorders.

## 1 Introduction

Schizophrenia is a common and complex psychiatric disorder with overall symptoms (mainly including negative, positive, and emotional symptoms) that may related to deficits in cognitive function (1–3). Currently, antipsychotic medication is the main treatment for schizophrenia, but cognitive impairment and some clinical symptoms may persist after medication (4), so it is urgent to find suitable psychosocial adjunctive therapies to improve overall symptoms and cognitive impairment. Cognitive remediation (5, 6), physical exercise (7), and music therapy (8) have been widely chosen. Music therapy has attracted much attention because of its unique and fun nature, and studies using it to improve clinical symptoms in schizophrenic patients have been reported many times (9, 10).

Music therapy is a psychosocial therapy method in which therapists use various experiences of music and the therapeutic relationship as a therapeutic motive to help the patients recover health (11). Existing studies have shown that music therapy conducted in individual or group form can improve overall symptoms and cognitive functions, such as attention and working memory in schizophrenia (12–16). However, due to the prevalence of symptoms such as social withdrawal in patients with schizophrenia, group music therapy is more widely used. It builds on the cooperation of the patient-patient group that requires group members to collaborate on tasks related to music therapy, helping schizophrenic patients establish social connections with the therapist and other group members, enhancing social cognitive abilities, and improving overall symptoms and cognitive function (17, 18).

EEG as an important tool for understanding neurological disorders, is mainly used to study abnormal neural oscillations in schizophrenic patients. Neural oscillations underlie the cognitive function of the brain and are considered a biomarker of neuropsychiatric disorders (19). Various studies have found that schizophrenic patients have abnormal low- and high-frequency oscillations, especially in theta and gamma bands, compared to healthy individuals (20–22). Higher theta oscillation in schizophrenia is associated with more severe symptoms (23) and poorer verbal memory performance (24). In addition, with the help of psychiatry and neurocognition scales, researchers have demonstrated that the decrease in gamma oscillation of schizophrenia is related to cognitive deficits such as distraction and memory impairment (25, 26). Thus, theta and gamma may be potential biomarkers for patients with schizophrenia.

Notably, the improvement of overall symptoms of music therapy in schizophrenia has been demonstrated by clinical behavioral evidence (27, 28), but the neuroimaging evidence is still insufficient. Therefore, we designed a five-week group music therapy for patients with schizophrenia who participated in our experiment and we explored the neural mechanisms of music therapy on the overall symptoms of schizophrenia with the help of MS (29) scores, PANSS scores, and EEG data.

## 2 Materials and methods

The study was done with the approval of the Ethics Committee of the University of Electronic Science and Technology of China (No. 1061420210305026). All procedures were carried out in adherence to approved guidelines. Written informed consent was obtained from all participants before the study.

### 2.1 Participants

Patients with schizophrenia recruited in this experiment were from Chengdu Dekang Hospital. Inclusion Criteria: 1) Meet the diagnostic criteria of schizophrenia in the Diagnostic and Statistical Manual of Mental Disorders, Fifth Edition, Text Revision (DSM-5-TR) (30), 2) aged between 20-70 years, 3) elementary school education or above, normal speech ability, normal communication, able to understand and complete the scale, Mini-Mental State Examination (MMSE) score ≥ 24, Montreal Cognitive Assessment (MoCA) score ≥ 25, 4) duration of the disease ≥ 5 years, hospitalization ≥ 6 months, and stable disease condition, 5) informed and consenting to participate. We divided the 38 patients who met the inclusion criteria into the music group and the visual art group. 2 of them were excluded because they voluntarily withdrew from the experiment early due to sudden health issues and family relocation, and 4 because their EEG data were of insufficient quality. The final analysis included 32 individuals. There were 17 participants included in the music group (age range 20-70 years, 10 female), and 15 participants in the visual art group (age range 20-70 years, 7 female). The two groups have similar socio-demographic data and clinical characteristics, including gender, age, marital status, education level, duration of illness, number of antipsychotics, MMSE score, and MoCA score (**Table 1**).

**Table 1:** Sociodemographic and clinical characteristics of study participants.

Characteristic	Music group	Visual art group	*P* value
Gender^a^ (male/female)	7/10	8/7	0.49
Age^b^ (years)	46.12 ± 15.22	47.60 ± 9.44	0.75
Marital status^a^ (married/unmarried)	1/16	1/14	0.93
Education level^b^ (years)	7.18 ± 4.91	6.60 ± 1.20	0.67
Duration of illness^b^ (years)	21.06 ± 12.83	16.93 ± 6.45	0.28
Number of antipsychotics^b^	1.94 ± 0.80	1.47 ± 0.72	0.10
MMSE score^b^	27.88 ± 1.23	28 ± 1.46	0.81
MoCA score^b^	26.53 ± 1.33	27 ± 1.32	0.34

Values are shown as mean ± SD, unless otherwise noted. P values refer to significance between two groups.

a: Chi-square test was used.

b: Two-sample t-tests was used.

### 2.2 Study design

During the five-week therapy process, the professional therapist who was certified as a U.S. registered Neurological Music Therapist (NMT) and Musical and Imagery Therapist (MIT) conducted twice-weekly 45-minute sessions for the music group. There were 10 topic courses in music therapy based on the aim of assisting patients to develop social skills and express their emotions. Therapy progress began with an assessment to give the therapist a better understanding of the musical ability of the patient. In each course, the therapist would set course activities that integrate singing, songwriting, and listening according to the course targets and ask patients to work together to complete these activities. Every course was divided into 3 or 4 stages, the first stage began with a chorus and then everyone sang their self-introduction song in turn, the activities of the second and third stages were decided by the course target, and the last stage of each course, patients get together to give a chorus named ‘Goodbye song’ (**Table 2**). Meanwhile, the visual art group learned basic handicraft and painting skills, including independently completing painting works, weaving handbags, or creating bookmarks, notebooks, and other activities.

**Table 2:** The music therapy schedule.

Course topics	Course targets	In-course activities			
		Stage 1	Stage 2	Stage 3	Stage 4
Assessment	Musical proficiency assessment	Hello song, self-introduction song	Warm-up activity	Instrumental ensemble	Goodbye song
Music experience	Emotional feelings and picture perception of songs		Music sharing	Music experience	
Musical figuration	The perception of musical emotions is concrete and substantial		Music collage	Music creation of the four seasons	
Music speed and mood	The speed of the music matches the emotion, and the body language of the emotion is expressed		Matching music speed and mood		
Music composition	Music and emotional expression		Music creation		
Re-assessment	Assessment of musical emotional perception		Music sharing	Music handicraft	
Music for self-care	The ego mood matches the musical mood		Music self-care		
Happy mood music	Positive emotions match the music		Happy music session	Music painting	
Sad mood music	Negative emotions match the music		Sad music session	Music handicraft	
End music album	Mood music matching		Mood music album	Music painting	

### 2.3 Data recording and processing

We recorded EEG signals and behavioral data. The professional doctor evaluated the severity of the clinical symptoms of patients using MS and PANSS scales based on their clinical manifestations before and after intervention. The resting EEG signals were recorded using the actiCHamp plus 64ch electroencephalograph (BrainProducts, Germany) with a 31-lead electrode cap system (Fp1/2, F3/4/7/8, T7/8, Pz/3/4/7/8, Cz/3/4, Oz/1/2, FT9/10, FC1/2/5/6, TP9/10, CP1/2/5/6) according to the 10/20 international electrode placement system. The initial sampling rate was 500Hz, and 31 channels were recorded simultaneously. We collected 15-minute resting EEG from participants in two groups before and after the intervention.

EEG preprocessing was done by the toolbox ‘EEGLAB’ and the ‘zero-reference’ code, utilizing the Reference Electrode Standardization Technique (https://www.neuro.uestc.edu.cn/name/shopwap/do/index/content/96). A Finite Impulse Response (FIR) band-pass filter (0.5-100Hz) was applied and the sampling rate was reduced to 100Hz. Independent Component Analysis (ICA) was used to remove ocular artifacts, following which the data was segmented into 2-second intervals, with a threshold of ±100 UV selected to exclude other artifacts.

Power spectral density (PSD), which describes the power distribution of signals in the frequency domain, is widely used to analyze the neural oscillation of schizophrenia (26, 31, 32). We calculated the theta and gamma power spectral densities using Welch’s method of the two groups at pre- and post-therapy.

### 2.4 Statistic analysis

IBM SPSS Statistics 26.0 was used to perform the statistical analysis. Numerical variables were given as means (standard deviation, SD), while categorical variables were described as numbers. The Chi-square (χ2) test was used for categorical variables, and the Student’s t-test or Mann-Whitney test was used for continuous variables according to their normal distribution and variance homogeneity. We compared the changes in PSD within the group using the paired t-test. Subsequently, to observe the difference in intervention effect between the two groups, we performed the two-sample t-test on the PSD. Besides, to explore the relationship between changes in theta and gamma oscillations and the improvement of overall symptoms in the music group, Pearson’s correlation analysis between the PSD and MS/PANSS scores was used.

## 3 Results

**Figure 1:**
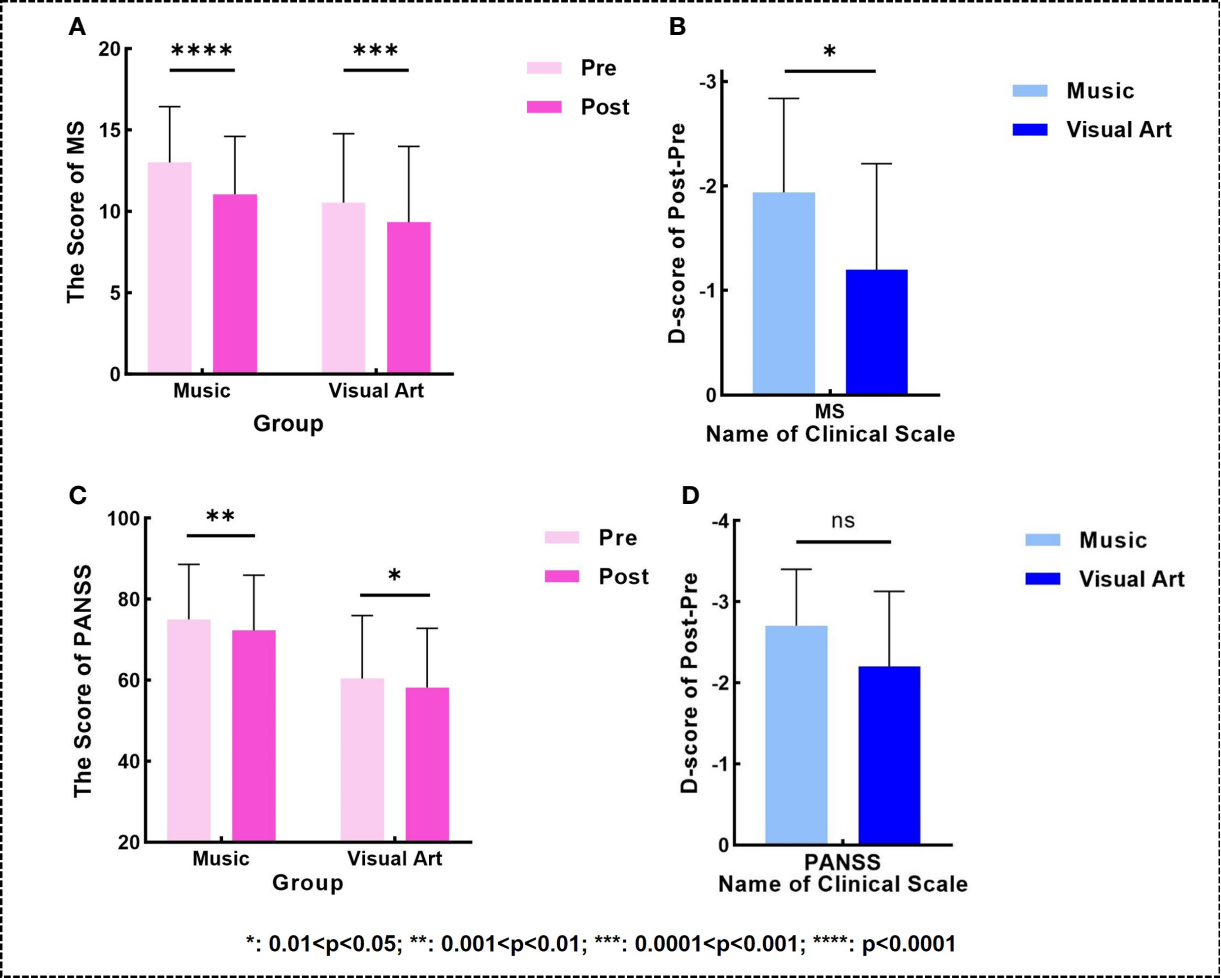
**(A, C)** Changes in the MS/PANSS scores (higher scores indicating more severe overall symptoms) within two groups. **(B, D)** comparison of post-pre differences in the MS/PANSS scales between two groups.

After the intervention, the MS and PANSS scores of the two groups significantly decreased (music group: *p_MS_* < 0.0001, *t_MS_* = -8.899, *p_PANSS_* = 0.0013, *t_PANSS_* = -3.891; visual art group: *p_MS_* = 0.0004, *t_MS_* = -4.583, *p_PANSS_* = 0.0325, *t_PANSS_* = -2.374; **Figures 1A, C**). Moreover, the decline in MS scores in the music group was greater than that in the visual art group (*p* = 0.0363, *t* = 2.192; **Figure 1B**). However, no significant between-group difference was observed in the decrease in PANSS scores (**Figure 1D**).

**Figure 2:**
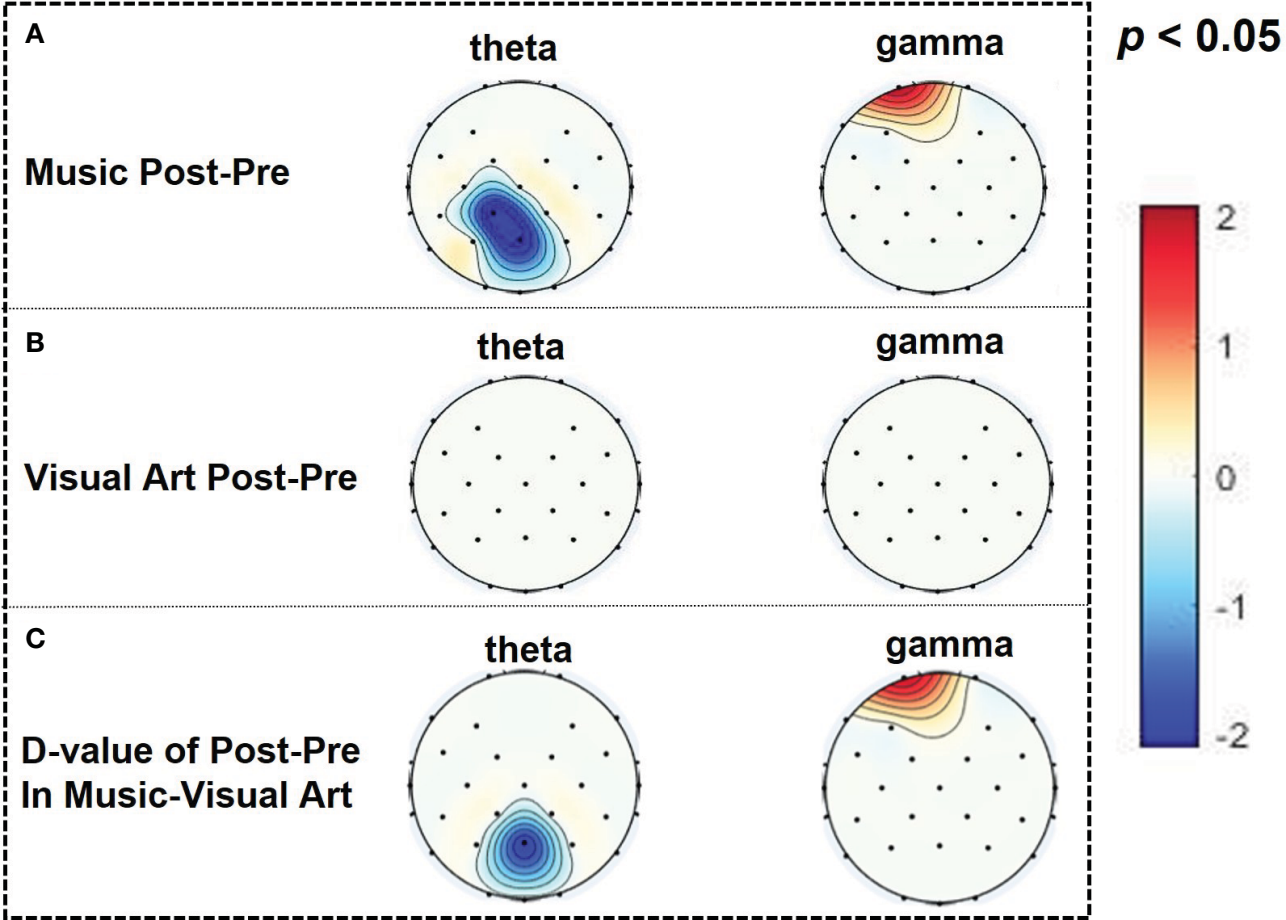
**(A)** The difference of post-pre PSD in theta (5-7Hz) and gamma (30-45Hz) bands within the music group (t-plot) and **(B)** the difference of post-pre PSD in theta and gamma bands within the visual art group (t-plot). **(C)** Comparison of post-pre PSD differences between two groups.

**Figure 3:**
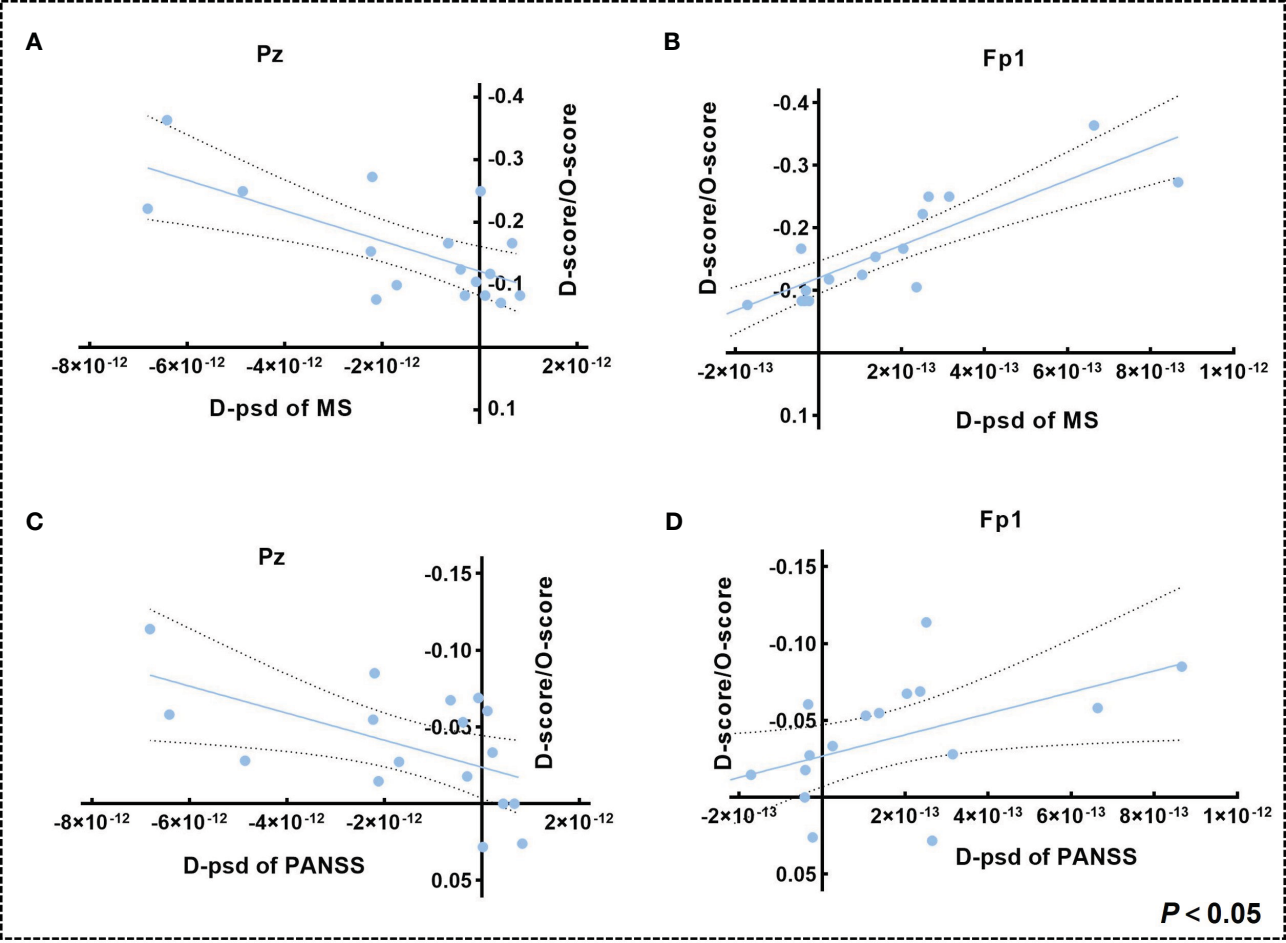
**(A, B)** Correlation between (The change of MS score/Original MS score) and the change of theta PSD in the parietal lobe and gamma PSD in the prefrontal lobe in the music group. **(C, D)** Correlation between (The change of PANSS score/Original PANSS score) and the change of theta PSD in the parietal lobe and gamma PSD in the prefrontal lobe in the music group (dashed intervals indicate 95% confidence intervals for the best-fit straight line).

In the music group, the parietal theta PSD decreased and the prefrontal gamma PSD increased (CP1: *p* = 0.0148, *t* = -2.7314; Pz: *p* = 0.0208, *t* = -2.5651; Fp1: *p* = 0.0480, *t* = 2.1415; **Figure 2A**), while there was no significant difference in the visual art group (**Figure 2B**). Subsequent results on the difference of post-pre PSD between the two groups showed that the music group has a significant decrease in theta PSD in the parietal lobe and an increase in gamma PSD in the prefrontal lobe compared with the visual art group (Pz: *p* = 0.0465, *t* = -2.0762; FP1: *p* = 0.0478, *t* = 2.0640; **Figure 2C**). Pearson’s correlation analysis results showed that changes in MS and PANSS scale scores were linearly correlated with the change of theta PSD in the parietal lobe and gamma PSD in the prefrontal lobe (Pz: *p_MS_* = 0.0025, *r_MS_* = 0.6837, *p_PANSS_* = 0.0225, *r_PANSS_* = 0.5490; Fp1: *p_MS_* < 0.0001, *r_MS_* = -0.8606, *p_PANSS_* = 0.0375, *r_PANSS_* = -0.5078; **Figure 3**).

## 4 Discussion

Consistent with previous studies (33, 34), we found that both music and visual art interventions can effectively help patients with schizophrenia relieve their overall symptoms. However, the decline in MS scores was greater in the music group than in the visual art group, and a trend-level effect observed for PANSS scores also favored the music group. This may be because compared with visual art intervention, which involves independent handicraft and painting activities, music therapy can provide a more interactive and collaborative therapeutic environment for patients with schizophrenia, helping them to become more engaged and build social connections (35, 36), alleviating negative and positive symptoms (9, 37), thereby improving their overall symptoms more effectively.

Previous studies have shown that theta and gamma oscillations appear to be associated with a wide range of perceptual and cognitive processes of schizophrenia (38, 39). We found that music therapy significantly decreased the theta oscillation in the parietal lobe and increased the gamma oscillation in the prefrontal lobe, analogous to the effects of antipsychotics or deep-brain stimulation (40–42), which may indicate the therapeutic effect of music therapy as a psychosocial intervention for patients with schizophrenia. These changes in theta and gamma oscillations in brain regions associated with cognitive function may reflect greater attention and better verbal memory capacity (24, 43–45). Patients with schizophrenia whose verbal memory capacity significantly declined manifested an increase of theta oscillations in the parietal lobe (45, 46). After taking medication, theta oscillations decreased (41). Our results are also consistent with these studies. In addition, schizophrenic patients with attention deficits are prone to symptoms such as fantasies and cognitive disorders, which may be related to their lower gamma oscillations (47, 48). Increased prefrontal gamma oscillations may be associated with the fact that musical rhythms and melodies increase patients’ interest and enhance auditory attention (49). Notably, the existing studies have shown that the presence of an active and trained therapist has an important influence on cognitive function in patients with schizophrenia (5, 50). In this paper, patients in the music group accepted a five-week therapy under the guidance of a professional music therapist who was certified as a U.S. registered Neurological Music Therapist and Musical and Imagery Therapist. This therapy was effective in improving theta and gamma oscillations, which may indicate a positive impact on cognitive performance but this must be further demonstrated by specialized measurements.

Correlation results showed that a decrease in parietal theta oscillations and an increase in prefrontal gamma oscillations were positively correlated with changes in the PANSS and Manchester scales. That is to say, after the music therapy, patients experienced a reduction in positive symptoms, negative symptoms, depressive symptoms, and anxiety symptoms, as well as a recovery in theta and gamma oscillations. These results suggested that these two types of neural oscillations may be bio-indicators to improve overall symptoms in schizophrenic patients and may also be targets for intervention.

## 5 Limitations

This study has some limitations. Firstly, the sample size of our participants was small, and future studies could increase the sample size to improve the reliability of the study. Secondly, further long-term follow-up studies could be conducted to assess the lasting effects of music therapy. Thirdly, we have not explored the neurological mechanisms underlying certain specific symptoms, which could be further investigated in the future to provide better music therapy for schizophrenics with specific symptoms. Fourthly, our assessment of overall symptoms relies on subjective measurement tools such as the Manchester and PANSS scores. Although these tools are widely used in psychiatric research, there are problems with subjective assessments, including the influence of patient expectations and the therapeutic relationship. Multiple assessment tools, including objective physiologic indicators and the establishment of a positive therapeutic relationship, should be used in future studies to gain a more comprehensive understanding of the patient’s symptomatic status and to improve the accuracy and credibility of findings.

## 6 Conclusion

Our study provides evidence that music therapy can be an effective adjunctive therapy to help patients with schizophrenia improve overall symptoms. This improvement may be related to the fact that music therapy helps patients with schizophrenia to establish social connections and increase engagement, which in turn alleviates overall symptoms. The EEG results indicated that music therapy can improve abnormal neural oscillations in patients with schizophrenia which is reflected by a decrease in theta oscillation in the parietal lobe and an increase in gamma oscillation in the prefrontal lobe. In addition, both reductions in theta oscillations in the parietal lobe and increases in gamma oscillations in the prefrontal lobe were positively correlated with the improvement of overall symptoms, suggesting that music therapy may improve overall symptoms by modulating neural oscillations in theta and gamma. Our findings may help us better understand the neural mechanisms of music therapy for schizophrenia and provide more evidence for the application of music therapy in other psychiatric disorders.

## Data availability statement

The raw data supporting the conclusions of this article will be made available by the authors, without undue reservation.

## Ethics statement

The study was done with the approval of the Ethics Committee of the University of Electronic Science and Technology of China (No. 1061420210305026). The studies were conducted in accordance with the local legislation and institutional requirements. The participants provided their written informed consent to participate in this study. Written informed consent was obtained from the individual(s) for the publication of any potentially identifiable images or data included in this article.

## Author contributions

LW: Conceptualization, Data curation, Writing – original draft, Writing – review & editing. LW: Methodology, Writing – original draft, Writing – review & editing, Investigation. JC: Investigation, Methodology, Writing – original draft. CQ: Writing – review & editing. TL: Data curation, Resources, Writing – review & editing. YW: Project administration, Resources, Writing – review & editing. YL: Investigation, Methodology, Writing – review & editing. PZ: Visualization, Writing – review & editing. SG: Conceptualization, Writing – review & editing. JL: Conceptualization, Data curation, Methodology, Writing – review & editing, Writing – original draft.
